# Effect of a Nutritional Education Intervention on Sports Nutrition Knowledge, Dietary Intake, and Body Composition in Female Athletes: A Pilot Study

**DOI:** 10.3390/nu17152560

**Published:** 2025-08-05

**Authors:** Macarena Veloso-Pulgar, Andreu Farran-Codina

**Affiliations:** 1 Departament de Nutrició, Ciències dels Aliments i Gastronomia, Facultat de Farmàcia i Ciències de l’Alimentació, Universitat de Barcelona, 08921 Santa Coloma de Gramenet, Spain; macarenaveloso87@ub.edu; 2 Institut de Recerca en Nutrició i Seguretat Alimentària (INSA-UB), Universitat de Barcelona, 08921 Barcelona, Spain

**Keywords:** nutrition education, female athletes, dietary intake, nutritional knowledge, body composition

## Abstract

Background/Objectives: Studies have reported that female athletes often exhibit low levels of nutritional knowledge and inadequate dietary intake to meet their nutritional needs. The aim of this study was to evaluate the effect of a nutritional education intervention on nutrition knowledge, dietary intake, and body composition in female handball players (*n* = 45; age, 17.6 ± 2.1 years). Methods: A quasi-experimental intervention design was implemented, consisting of a 3-week educational program delivered through six in-person sessions led by a registered dietitian. Nutrition knowledge, dietary intake, adherence to the Mediterranean diet, and anthropometric and body composition measurements were assessed. Results: Nutrition knowledge levels were significantly higher both immediately post-intervention and three months later compared to baseline (*p* < 0.05, ES > 0.8). A total of 36 participants completed a 3-day dietary record at baseline and at follow-up. Initial assessments revealed insufficient energy (31 kcal/kg/day) and carbohydrate intake (3.0 g/kg/day) and a high intake of total fats (1.4 g/kg/day). During follow-up, a significant decrease in the consumption of foods rich in sugar was observed (*p* = 0.0272). A total of 82.2% of the players needed to improve their adherence to the Mediterranean diet. No significant changes were found in Mediterranean diet adherence or body composition following the intervention. Conclusions: The nutritional education intervention significantly improved athletes’ nutritional knowledge and significantly decreased their consumption of sugary foods; however, further studies are needed to evaluate its impact on dietary intake and body composition, considering the study’s limitations.

## 1. Introduction

A balanced diet, both in terms of quantity and quality, is essential for replenishing energy stores and providing the necessary nutrients for the optimal functioning and efficient recovery of body systems following physical activity [1]. Accordingly, proper nutrition not only enhances athletic performance but also contributes to the prevention of fatigue, injuries, and other potential health issues in athletes [2].

Food choices are influenced by a variety of factors, including those related to the food environment, such as product availability and cost, as well as individual socioeconomic conditions and the availability of family and community resources that determine the affordability of nutritional recommendations [3]. In addition, food literacy, defined as the combination of knowledge, skills, and behaviors required to plan, manage, select, prepare, and consume food appropriately, plays a key role in shaping dietary intake [3].

Improving athletes’ nutrition knowledge may enhance their ability to make informed decisions. Greater nutritional knowledge enables individuals to understand the impact of daily food choices on performance, recovery, and overall health. This understanding supports adherence to nutritional recommendations and promotes the adoption of healthy eating habits. Existing evidence indicates that higher levels of sports nutrition knowledge are associated with improved dietary behaviors among athletes [4,5,6,7].

Female athletes undergo growth and development processes that differ from those of males, leading to significant differences in body size, composition, and hormonal profiles [8]. Moreover, sex-based differences have been observed in mitochondrial function, substrate utilization, immune responses, iron metabolism, thermoregulation, hydration, appetite control, energy availability, and endocrine functioning [9]. These physiological characteristics expose female athletes to specific nutritional and health challenges, especially in the context of intense physical activity, which may affect their overall well-being [10].

Numerous studies have identified inadequate energy intake among female athletes, compromising the fulfillment of nutritional requirements, particularly for carbohydrates and essential micronutrients such as calcium, iron, and some vitamins [11,12,13]. This situation may be related to disordered eating behaviors, often driven by social pressures, athletic expectations, and the construction of athletic identity [14]. Persistent energy deficits can result in low energy availability (LEA), a condition that may progress to the female athlete triad, characterized by the coexistence of LEA, osteoporosis, and functional hypothalamic amenorrhea [15].

Among the barriers that hinder improvements in nutrition knowledge and awareness among athletes are the lack of access to evidence-based professional advice and practical nutrition education [15,16]. Several studies have reported insufficient levels of nutritional knowledge among female athletes [11,12,13,17,18]. These findings may be due to the limited implementation of targeted educational programs. This situation is often aggravated by exposure to contradictory and unreliable information from non-specialist sources, such as family members, coaches, peers, or the internet, which may create confusion and make it difficult to adopt appropriate eating habits [19].

The Mediterranean diet is characterized by a high content of antioxidants and anti-inflammatory compounds, including vitamins, folates, flavonoids, and omega-3 fatty acids [20]. This composition contributes to its well-documented health benefits, such as reduced cardiovascular risk, improved metabolic health, overall well-being, and positive effects on athletic performance [21]. Among athletes, adherence to this diet has been associated with enhanced recovery, reduced inflammation, and support for training adaptations [22]. Therefore, promoting adherence to the Mediterranean diet is a relevant goal in nutritional education programs.

Nutritional education interventions aim to improve knowledge and dietary behaviors through structured programs [23]. Given the nutritional risks faced by female athletes, there is growing interest in evaluating the impact of such programs in this population and expanding this research in diverse sports contexts, age groups, and levels of competition [10]. In this context, the primary aim of the present study was to evaluate the effect of a nutritional education intervention on sports nutrition knowledge, dietary intake, and body composition in female athletes, as well as to identify factors that may influence the effectiveness of the intervention. We hypothesized that the nutritional education intervention would significantly improve sports nutrition knowledge and dietary intake and would lead to favorable changes in body composition in female athletes.

## 2. Materials and Methods

### 2.1. Study Design

**Figure 1:**
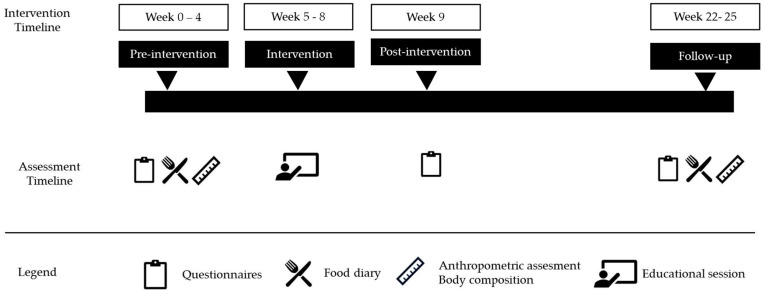
Setting and schedule of the intervention.

This pilot study adopted a quasi-experimental intervention design. A three-week nutritional education program was implemented with a group of female handball players. The study protocol complied with the principles outlined in the Declaration of Helsinki for research involving human participants and was approved by the Bioethics Committee of the University of Barcelona (IRB00003099) on 30 June 2023. All players, along with their parents and coaches, received detailed information about the study, both orally and in writing. Participation was voluntary and required the signing of an informed consent form by the players or by their parents in the case of participants under 16 years of age. The study was conducted between September 2023 and October 2024 and was structured into four phases (Figure 1): pre-intervention, intervention, post-intervention, and follow-up (conducted three months after the end of the intervention).

### 2.2. Subjects

Participant recruitment was conducted at a sports club and a high-performance center located in the province of Catalonia (Spain) during September 2023 and March 2024, respectively. An initial meeting was held with the technical directors and coaches of both institutions to present the project and invite their participation. Subsequently, the project was introduced to the players in a separate session with the same objective.

The inclusion criteria were (a) female players aged between 14 and 24 years, (b) with at least one year of experience training at the club, and (c) training at least three days per week for approximately 1.5 h per day. Exclusion criteria included (a) diagnosis of an eating disorder, (b) presence of a chronic condition requiring a specific dietary plan, (c) pregnancy, and (d) inability to speak Spanish.

### 2.3. Intervention

Before the intervention, all participants were called to a 60 min meeting to learn about their interests in nutrition and their expectations in improving their dietary habits. The nutrition education intervention consisted of six face-to-face group sessions of 15 min each, delivered twice a week. Attendance at each session was recorded. The contents covered are presented in Table 1. Each session was designed and delivered by a registered dietitian-nutritionist.

At the end of the sessions, the coaches were sent, by e-mail, the visual material used in each meeting, together with the guide entitled “Nutrition for Young Female Athletes”. This document, prepared by a registered dietitian in collaboration with final-year students of the Degree in Human Nutrition and Dietetics at the University of Barcelona, included theoretical information and practical examples to improve nutrition for sports performance. The coaches were responsible for distributing this guide to the players and their families.

Several factors were considered to maximize the achievement of the proposed objectives. The location and schedule of each meeting were arranged according to the players’ availability in order to facilitate attendance. The topics covered were adapted and, in some cases, selected according to the participants’ prior knowledge and interests, allowing the content to be tailored to their actual needs.

### 2.4. Data Collection and Measurements

#### 2.4.1. Questionnaires

The questionnaires were self-administered and completed online using forms developed with the Open Data Kit tool on the Kobo Toolbox platform (European server). Participants were informed that all collected data would be treated as confidential.

Socio-demographic questionnaire

Basic personal and demographic information was collected through a brief questionnaire developed by the research team. This questionnaire included variables such as age, competition category, educational level, years of training, employment status, and maternal educational level, among others. The questionnaire was administered at the beginning of the study, and completion of it was a mandatory requirement for the player’s final inclusion in the research.

Sports nutrition knowledge questionnaire

Nutritional knowledge was assessed using the previously validated Sports Nutrition Knowledge Questionnaire for Young and Adult Athletes (NUKYA) [24]. The NUKYA questionnaire consists of 20 questions comprising a total of 59 items that evaluate nutritional knowledge across four domains: macronutrients, micronutrients, hydration, and periodization (food and fluid intake before and during exercise). The maximum possible scores (out of 100) for each section were as follows: macronutrients, 49.1 points; micronutrients, 32.2; hydration, 13.6; and periodization, 5.1. The questionnaire was administered during the pre-intervention, post-intervention, and follow-up phases. It was conducted under the supervision of the principal investigator to prevent discussion and information exchange among respondents.

Eating Attitudes Test

At the beginning of the study, the Eating Attitudes Test (EAT-26) [25] was used to assess the risk of eating disorders. It consists of 26 items grouped into three subscales: dieting, bulimia, and food preoccupation. Responses to each item are measured on a 6-point Likert scale. A total score of 20 or higher indicates a risk of an eating disorder.

Body Shape Questionnaire

At the beginning of the study, the Body Shape Questionnaire (BSQ) [26] was used to assess body image perception and dissatisfaction with body shape and weight. The questionnaire consists of 14 items related to self-image, which are rated on a 6-point Likert scale ranging from 1 (never) to 6 (always). A total score is obtained by summing the item scores, resulting in a possible range from 14 to 84. Higher scores indicate greater dissatisfaction with body shape and weight.

#### 2.4.2. Adherence to the Mediterranean Diet

Adherence to the Mediterranean Diet (MD) was assessed using the Kidmed index [27], which comprises a 16-question test. Questions with a negative connotation regarding the MD were assigned a value of −1, while those with a positive aspect were assigned +1. The scores from the 16 items were summed to classify the diet into three levels: ≤3 indicating very poor diet quality; 4–7 indicating the need for improvement to align intake with Mediterranean patterns; and ≥8 indicating optimal adherence to the MD. Adherence was evaluated at the pre-intervention phase and at follow-up.

#### 2.4.3. Assessment of Dietary Intake

A 3-day dietary record supported by images and a mobile application was collected during the pre-intervention and follow-up phases. The record included two non-consecutive weekdays and one weekend day, which corresponded to the match day.

Prior to data collection, participants were invited to an in-person meeting where detailed instructions were provided regarding the recording procedure and the importance of accurate dietary reporting. First, participants were asked to download the Remind^®^ mobile application (version 15.2) [28]. Then, they received training on how to use the app and take photographs that accurately represented their food intake. Participants were instructed to photograph all foods and beverages consumed at a 45° angle using a fiducial marker (a reference object with known dimensions), such as a pen or cutlery [29]. They were also instructed to take photos of second servings and leftovers. In addition to submitting photographs via the app, participants were asked to provide a brief written description of the foods consumed and the time of consumption. The Remind^®^ app allows for real-time communication, enabling researchers to monitor participant progress, send reminders to submit photographs, and request any missing information. All the dietary records were reviewed daily by final-year undergraduate students in Human Nutrition and Dietetics at the University of Barcelona, who received previous training to ensure proper handling and assessment of dietary record data and to homogenize the criteria used during data processing.

Image-based dietary data were analyzed by a registered dietitian using the PCN Pro 1.0 software [30]. This software uses composition data from the Spanish Standard Food Composition Tables (TECA) [31].

To estimate the portion sizes of foods consumed, photographic portion guides based on commonly consumed foods in Spain were used [32,33]. Subsequently, the average daily intake of energy (kcal/day), macronutrients (g/day), and micronutrients (mg/day or µg/day) was calculated. The nutrients selected for analysis were those considered critical for female athletes: calcium, magnesium, iron, zinc, folate, vitamin C, vitamin D, vitamin E, vitamin B6, and vitamin B12. In addition, the average daily intake (g/day) of the food groups presented in Table 2 was estimated.

#### 2.4.4. Anthropometric Measurements and Body Composition

Anthropometric and body composition measurements were conducted during the pre-intervention and follow-up phases. The pre-intervention measurements were carried out during the competitive phase, while the follow-up measurements took place during the sports preparation phase. Body weight and height were measured using a mechanical column scale with a stadiometer (Model 711 with 220 cm height, 0.5 cm precision; Seca, Hamburg, Germany). Body composition, including fat mass (FM), muscle mass (MM), and fat-free mass (FFM), was assessed using a multifrequency bioelectrical impedance analyzer (BIA, Z-Metrix Bioparhom, France).

For all measurements, participants were required to attend the session in the morning, wearing only underwear and following an overnight fast of at least 8 h. For the assessment of body composition, participants were instructed to refrain from consuming coffee, tea, or other sources of caffeine on the day of the measurement; to avoid strenuous physical activity for at least 12 h beforehand; and to urinate prior to the evaluation. All assessments were conducted by a registered dietitian at the respective training centers.

### 2.5. Statistical Analysis

Responses to the NUKYA questionnaire were entered directly into a database and subsequently scored as follows: a score of 0 was assigned to the options ‘not sure/don’t know’, a positive score of 1 to correct answers, and a negative score of −1 to incorrect answers. The scores for each item were summed up to obtain both the total score and the score for each section of the NUKYA questionnaire. These scores were then divided by the maximum possible score in each case to calculate percentages. Because incorrect answers were penalized in the scoring system, negative scores were possible.

Normality of the data was assessed using the Shapiro–Wilk test, and homoscedasticity was evaluated using Levene’s or Bartlett’s test for equal variances. Means were compared using Student’s *t*-test or, alternatively, the Mann–Whitney test. Changes in NUKYA scores before the educational intervention, after the intervention, and at follow-up were analyzed using a linear mixed-effects model (LMM), with post hoc pairwise comparisons adjusted using the Bonferroni method. This approach was preferred over repeated measures ANOVA due to the presence of some missing values, and prior to the analysis, we verified that the missing values did not depend on any variable (missing at random, MAR). Effect sizes were calculated using Hedges’ g (very small effect < 0.2; 0.2 ≤ small effect < 0.5; 0.5 ≤ moderate effect < 0.8; large effect ≥ 0.8).

The strength of association between variables was assessed using Pearson or Spearman correlation analyses. Some relationships between variables were examined using analysis of covariance (ANOVA). For all analyses, the confidence level was set at 95%. Data analysis was performed using STATA statistical software (version 16.1, StataCorp, College Station, TX, USA). The significance level was set to *p*-value < 0.05.

For the analysis of nutritional knowledge, which was assessed across three time points (baseline, post-intervention, and follow-up) using linear mixed models, sample size estimation was performed with the GLIMMPSE online tool (General Linear Mixed Model Power and Sample Size; University of Colorado Anschutz Medical Campus, Aurora, CO, USA). Based on expected means and standard deviations derived from previous studies, and using the Hotelling–Lawley Trace test with an alpha level of 0.05 and power of 0.80, the minimum required sample size was estimated to be 24 participants. To estimate the minimum required sample size, a statistical power of 0.80, an alpha level of 0.05, and a medium effect size (Cohen’s d = 0.5) were assumed. Using G*Power software (version 3.1.9.4, Heinrich Heine University Düsseldorf, Düsseldorf, Germany), the minimum required sample size was calculated to be 27 athletes for the analysis of dietary intake (nutrient consumption), based on a paired *t*-test design. We estimated a 20% loss of subjects during the study, so the minimum number of subjects to be recruited was set at 32 athletes.

## 3. Results

### 3.1. Sample Characteristics

A total of 79 players were invited to participate (60 from the sports club and 19 from the high-performance center), of whom 52 were initially enrolled (33 and 19, respectively). Two players were finally excluded because they had been training for less than a year. The participants represented three competitive categories according to the “Real Federación Española de Balonmano” (Royal Spanish Handball Federation): “cadete” (<16 years), “juvenil” (<18 years), and “senior” (>18 years).

Of the 50 players recruited who signed the informed consent form, 5 were excluded for not completing the basic data questionnaire (4 “cadetes” and 1 “senior”). A total of 45 participants were ultimately included in the study. Their ages ranged from 14 to 23 years, with a mean age of 17.6 ± 2.1 years (Table 3). A total of 57.8% *(n* = 26) were members of the Club, while 42.2% (*n* = 19) belonged to the high-performance center.

In the EAT-26 assessment, the mean score was 5.8 ± 7.2. A total of 4.4% (*n* = 2) of the participants scored above 20 points, indicating a high risk of eating disorders. Regarding the BSQ test, the mean score was 30.0 ± 12.2. A total of 20.0% (*n* = 9) of the sample showed high dissatisfaction with their body image and weight.

### 3.2. Sports Nutritional Knowledge

Table 4 shows the changes in nutrition knowledge. Of the 45 players included in the sample, 44 completed the NUKYA questionnaire at baseline, 41 after the intervention, and 35 at follow-up. It was verified that the existing missing values were MAR, so LMM was applied to analyze changes in nutritional knowledge, using all the scores obtained from the players. A statistically significant increase of 20.3 percentage points was observed in the total questionnaire score between baseline and follow-up (*p* < 0.001), with a large effect size (ES > 0.8). The four domains evaluated also showed significant improvements (*p* < 0.005).

It is worth noting that scores increased substantially immediately after the intervention. Although a slight decline was observed in the total score and in most sections at follow-up compared to the post-intervention phase, this difference was not statistically significant.

### 3.3. Mediterranean Diet Score Adherence

At baseline, 17.8% (*n* = 8) of the sample demonstrated optimal adherence to the Mediterranean Diet (MD), while 68.9% (*n* = 31) required improvement to align their intake with Mediterranean dietary patterns. A total of 13.3% (*n* = 6) exhibited a very low diet quality. Thirty-six participants completed the Kidmed Index at both baseline and follow-up assessments. The mean baseline score was 5.8 (2.0), increasing slightly to 6.1 (1.7) at follow-up. However, this 0.3-point difference was not statistically significant (*p* = 0.2011).

### 3.4. Dietary Intake Assessment

Table 5 presents the changes in energy and macronutrient intake throughout the study. Analysis of three-day dietary records, completed by 36 participants at baseline and follow-up, revealed a significant decrease in fiber intake (*p* = 0.0169) and polyunsaturated fatty acids (*p* = 0.0006).

Regarding micronutrient intakes (Table 6), a significant decrease in vitamin E intake was observed (*p* = 0.0176).

When analyzing food group intake in grams per day, a significant decrease was observed in the consumption of sugar-rich foods (*p* = 0.0272, *n* = 36), from 21.3 (26.5) g/day at baseline to 13.0 (9.5) g/day at follow-up. When correlating the consumption of foods rich in sugar and attendance at educational sessions, correcting for age, a statistically significant relationship was observed (*p* = 0.0237). Regarding the consumption of fruits and vegetables, an increase was observed in both food groups during the follow-up phase. Total fruit and vegetable intake increased from 266.4 g/day (172.6) at baseline to 290.1 g/day (163.8) at follow-up; however, this difference was not statistically significant (*p* = 0.1683).

### 3.5. Anthropometric Measurements and Body Composition

Anthropometry was assessed in the baseline and follow-up phases in 35 of the 45 subjects who participated. Table 7 presents the anthropometric and body composition data observed in both study phases. A significant increase was noted in body weight (*p* = 0.0175) and BMI (*p* = 0.0161).

## 4. Discussion

The aim of this study was to evaluate the effect of a nutrition education intervention on nutrition knowledge, dietary intake, and body composition in female handball players. For this purpose, an in-person educational program was designed and implemented, led by a registered dietitian with expertise in the field. The intervention resulted in a significant increase in nutrition knowledge and a significant reduction in the daily consumption of sugar-rich foods.

### 4.1. Sports Nutritional Knowledge

Nutritional knowledge levels were significantly higher both immediately after the intervention and three months following its implementation, compared to baseline. This indicates that the three-week nutritional education intervention was effective in improving nutritional knowledge and that these improvements were sustained over time. Regarding the domains assessed by the NUKYA questionnaire, the items related to macronutrients and hydration showed the greatest increases in the post-intervention phase, with a large effect size (ES > 0.8). Other studies conducted in female athletic populations agree that nutritional knowledge is insufficient [34,35]. A study by Vázquez et al. [17], which assessed nutritional knowledge using the same questionnaire (NUKYA), reported similar scores (mean = 22.8; SD = 13.3) in a sample of 51 non-athlete females aged 14 to 16 years. These findings suggest that there are no substantial differences in nutritional knowledge between female athletes and non-athletes of the same age and that there is considerable room for improvement in their nutritional knowledge.

In general, studies evaluating the impact of nutritional education interventions in female athletes report significant improvements in nutritional knowledge [12,18,36,37,38,39,40,41,42,43,44,45,46,47,48,49]. A recent systematic review concluded that nutritional education interventions are effective in enhancing nutritional knowledge among female athletes [10]. However, the variability in the strategies used in terms of format, frequency, and duration makes it difficult to identify the specific characteristics an intervention should include to achieve the desired outcomes.

In our study, the intervention consisted of six in-person educational sessions held twice per week, totaling 90 min. This approach resulted in a significant increase of 22.0 percentage points in the total questionnaire score in the post-intervention phase. A study published by Chapman et al. [37], which implemented an educational intervention of the same duration, also reported a significant improvement in nutritional knowledge among female softball players (*p* < 0.01), with a score increase of 17.9 percentage points in the intervention group. These findings suggest that even a brief intervention, if well-structured and based on fundamental principles of sports nutrition, can lead to positive changes in nutritional knowledge.

When assessing the correlation between initial nutritional knowledge and age, we found that age was positively associated with higher nutritional knowledge (*p* = 0.0038), which agrees with other studies [50,51,52].

### 4.2. Mediterranean Diet Score Adherence

The Mediterranean diet (MD) is widely recognized as a healthy dietary pattern with benefits for both general health and athletic performance [21]. It is associated with higher nutritional quality, reduced disease risk [53], and improved physical outcomes such as muscular strength, endurance, and body composition [54]. These effects are attributed to its high content of antioxidants and anti-inflammatory compounds, including vitamins, folates, flavonoids, and omega-3 fatty acids [20,55,56]. Its balanced composition of complex carbohydrates, lean proteins, and unsaturated fats makes the MD an ideal framework for sports nutrition [57]. It can be used as a foundation for dietary planning, adapting to the individual energy and nutrient requirements of each athlete [58].

At baseline, 82.2% (*n* = 37) of the players exhibited dietary patterns requiring improvement, either due to moderate adherence or a low-quality diet. This finding aligns with Philippou et al. [58] and Sahnoune et al. [59], who reported that 100% and 90%, respectively, of female athletes assessed by the Kidmed Index required dietary improvements. These results highlight a common issue in this population: female athletes need to adjust their eating habits to align with current nutritional recommendations.

Unlike our findings, these studies reported significant post-intervention improvements in Kidmed scores. Notably, their interventions were longer in duration and employed diversified methodologies, such as workshops, which may have facilitated more effective dietary changes.

Similar trends have been reported in non-athlete Spanish adolescents, with a gradual departure from the MD characterized by reduced consumption of fruits, vegetables, legumes, nuts, and fish, and increased intake of ultra-processed foods and fast food [27,60]. This pattern was also evident among our participants, indicating a shift towards less healthy dietary habits.

### 4.3. Dietary Intake

Nutrition in young athletes is essential to optimize performance, recovery, and overall health. It must be tailored to the specific demands of each athlete based on training load, ensuring an adequate intake of energy, macronutrients, and essential micronutrients. In this context, the nutritional education intervention, although it did not result in significant changes in energy and macronutrient intake, did result in a significant reduction in the consumption of sugary foods. However, in the case of dietary fiber and vitamin E, changes have been unfavorable. It should be considered, in any case, that in the absence of a control group it is difficult to assess whether such observations may be related to the intervention or due to other factors such as seasonal changes in diet.

At the beginning of the study, the average energy intake was approximately 2000 kcal/day (31 kcal/kg of body weight/day). In other studies, conducted with female athletes, reported intakes were even lower, with values below 30 kcal/kg/day [11,12,47,48,61,62]. This situation is concerning, as insufficient energy intake has been shown to be frequently associated with deficiencies in various micronutrients in this population [63].

Carbohydrate intake was inadequate both at baseline and during the follow-up phase. Carbohydrates accounted for 40% of total energy intake, with an average consumption of 3.0 g/kg/day. These values fall below the current recommendations for athletes engaged in moderate physical activity, which suggest that carbohydrates should contribute at least 45% of total caloric intake, with an average consumption of 5–7 g/kg/day [64]. Insufficient carbohydrate intake may negatively affect athletic performance [65], highlighting the importance of educating athletes about adequate carbohydrate consumption. In the review published by Larrosa et al. [66], which included only studies conducted in female athletes, it was concluded that high-carbohydrate diets improve performance in activities that deplete muscle glycogen stores. Despite the overall low carbohydrate intake, a significant reduction in the consumption of sugary foods was observed after the intervention. This suggests a possible improvement in carbohydrate quality, as the athletes may have replaced processed foods high in sugar with more nutritious options. Furthermore, a positive correlation was found between attendance at the educational sessions and the decrease in the consumption of sugary foods, indicating that higher participation was associated with a greater reduction in these types of foods. This finding may be related to the emphasis placed during the sessions on the importance of reducing the intake of sugary and ultra-processed products.

Fiber intake (17.7 ± 5.4 g/day) was far below the current recommendations (25–35 g/day) [67]. These results are consistent with those observed in other studies [11,59].

In both assessment periods, protein intake aligned with current recommendations (1.2–2.0 g/kg/day) [64], with an average intake of 1.5 g/kg/day. This is in line with findings from other studies involving female athletes [11,12,18,59,68,69,70].

Regarding total fat intake, both assessment periods revealed excessive consumption, with fats contributing 40% of total energy intake and an average intake of 1.4 g/kg/day, while the recommended range is 20–35% of total energy intake [64]. A preference for high-fat foods over carbohydrate-rich options has also been reported in other studies [12,48,71]. It is important to note that current scientific literature does not support the hypothesis that extremely high-fat, carbohydrate-restricted diets enhance performance in competitive athletes [64,72]. Moreover, when analyzing the type of fats consumed, our results show that 14% of total calories came from saturated fatty acids, exceeding the recommended limit of less than 10% [64].

Regarding micronutrients, their intake is crucial to maximize the training potential of athletes [73]. However, due to inadequate dietary habits, especially if the intake is not adjusted to the requirements for physical activity, they are a population prone to consume insufficient amounts of essential micronutrients [74]. In the case of female athletes, it is common to find deficiencies of iron, calcium, and vitamin D [65]. After the implementation of the intervention, no significant increase was observed in any of the aforementioned micronutrients.

Regarding antioxidants, they play a crucial role in protecting against oxidative damage caused by exercise [64]. A diet rich in fruits and vegetables represents an effective strategy to ensure adequate antioxidant intake [75]. Although there was a trend toward increased total fruit and vegetable intake after the intervention, it remained insufficient, with an average consumption of 290.1 g/day (163.8). These values are similar to those reported by the National Food Consumption Survey in Children and Adolescents (ENALIA) conducted in Spain (2012–2014), which found an average daily intake of 267.1 g/day of fruits and vegetables [76]. These values fall well below the WHO recommendation, which establishes a personal minimum of 400 g/day [77].

### 4.4. Anthropometric Measurements and Body Composition

Maintaining an optimal body composition is crucial for athletic performance, as it enhances physical condition and reduces the risk of injuries [78]. Consequently, it is important to investigate whether nutritional education interventions can positively influence athletes’ body composition.

Our findings indicate that the participants’ body composition did not undergo significant changes following the intervention, a result consistent with previous research [49,59,61,69,70,79,80].

A systematic review by Veloso-Pulgar et al. [10], which examined the impact of nutritional education interventions on body composition in female athletes, found that only 3 out of 9 studies reported significant improvements, such as reductions in fat mass and increases in lean body mass [11,47,81]. Notably, these successful interventions included individualized dietary counseling sessions where professionals provided tailored guidance to each participant, a factor that may have facilitated greater adherence and effectiveness.

One possible factor influencing our results is that anthropometric and body composition measurements were conducted after a vacation period, probably with physical inactivity. This timing may have affected their habitual physical condition and contributed to the observed increases in body weight and BMI.

### 4.5. Limitations and Strengths

This pilot study presents several limitations that should be considered. First, the lack of a control group limits the ability to draw robust conclusions regarding the effectiveness of the implemented educational intervention. Second, although both the educational materials used in the group sessions and the dietary guide were provided to the players and their coaches, the coaches did not participate in the educational sessions. Considering that coaches are one of the main sources of nutritional information for athletes [17], and studies have indicated that they may have limited nutritional knowledge [82], their active inclusion in future interventions could enhance the overall impact of the program. Moreover, although the nutritional guide was provided to the players’ parents, they were not invited to participate directly in the educational sessions. It should be noted that, in most cases, parents are responsible for purchasing and preparing food at home; therefore, their involvement could be a key factor in supporting sustained changes in eating habits and improving the players’ dietary intake. However, in practice, coordinating their attendance at the scheduled sessions proved logistically challenging due to constraints such as work schedules, family responsibilities, and limited availability to attend at the designated times. Third, the inherent limitations of dietary records must be acknowledged. Although this method is widely used in dietary assessment within research contexts, self-reported food records are subject to bias, particularly underreporting of intake [83]. Fourth, the follow-up dietary assessment and anthropometric measurements were conducted immediately after an extended vacation period, coinciding with the sports preparation phase. During this time, players were exposed to a prolonged break from regular physical training and potentially more irregular eating habits, which may have influenced the results obtained.

Despite the limitations mentioned above, this study presents several strengths. First, it contributes to the body of research specifically focused on young female athletes, a group that remains underrepresented in the scientific literature despite their distinct nutritional needs. Secondly, several aspects of this study align with the recommendations of the Guidelines for Effective Nutrition Interventions and Education (GENIE) [84]. The program was appropriately described, including its purpose, clearly defined objectives, and detailed content. The intervention was designed and delivered by a registered dietitian, ensuring expert input from the outset and aligning with GENIE’s emphasis on involving qualified professionals. The evaluation process followed GENIE principles, incorporating validated tools such as NUKYA and the KIDMED index to assess the program’s objectives. Additionally, the educational content was adapted to the participants’ prior knowledge and specific interests, enhancing relevance and engagement. The face-to-face sessions held at the sports clubs also contributed to a supportive learning environment, encouraging interaction and discussion.

## 5. Conclusions

This study showed that female handball players initially exhibited low levels of nutrition knowledge and inadequate dietary intake. Following the implementation of a three-week educational intervention, a significant increase in nutrition knowledge was observed both immediately after the intervention and at follow-up. Regarding dietary intake, a significant decrease in the intake of foods high in added sugars was observed.

Although these findings support the importance of including nutrition education programs led by qualified professionals to improve both knowledge and dietary habits in athletes, the results should be interpreted with caution, as the study design did not include a control group, which limits the ability to draw firm causal conclusions.

It is recommended to continue researching educational strategies aimed at female athletes that not only promote increased nutritional knowledge but also achieve a true transformation in their eating habits, improving their intake of energy and essential macro- and micronutrients. This will allow for the design of more effective interventions tailored to their specific needs, with the goal of maximizing their impact on health and athletic performance.

## Figures and Tables

**Table 1:** Topics covered in the educational sessions.

Session	Topic
1	Introduction to health and the Mediterranean diet
2	Energy and carbohydrate requirements
3	Protein and fat requirements
4	Micronutrients
5	Hydration
6	Supplementation and periodicity

**Table 2:** Food groups used to aggregate data on food consumption, with a short description of the foods included in each group.

Food Group	Foods
Fruits	Fresh fruits, canned fruits, and dried fruits
Vegetables	Leaf, flower, or stem vegetables, root vegetables, bulbs, and mushrooms
Cereals and grains	Cereals, grains and flour, pasta, baked goods, cookies, pastries, and breakfast cereals
Legumes	Legumes, dry legumes, legume flour, and derivatives
Tubers	Potatoes and other starchy tubers
Milk and dairy products	Milk and milkshakes, yogurt and fermented milk, dairy desserts, fresh cheese, aged cheese, processed cheese, milk ice cream, or similar
Meats	Pork, veal, lamb, beef, rabbit, poultry, viscera, and raw, raw-cured, and heat-treated sausages
Eggs	Chicken eggs and other eggs from other birds
Fish	Cod, hake, salmon, tuna, sole, monkfish, mackerel, sardines, etc.
Oils and fats	Olive oil, sunflower oil, coconut oil, lard, butter, and margarine
High-sugar foods	Sugar, honey, syrups, jams, chocolates, and candies
Nuts	Almonds, walnuts, hazelnuts, cashews, pistachios, macadamia nuts, pecans, and peanuts

**Table 3:** Demographic characteristics of participants at baseline (*n* = 45).

	*n*	%
Competition category		
Cadetes	14	31.1
Juvenil	14	31.1
Senior	17	37.8
Team rol		
Player	39	86.7
Goalkeeper	6	13.3
Training period (years)		
1–5 years	13	28.9
>5 years	32	71.1
Level of education		
Basic	16	35.6
Intermediate	16	35.6
University	13	28.8
Currently employed		
Yes	6	13.3
No	39	86.7
Mother’s highest educational level		
Without studies or only compulsory education	5	11.1
Secondary education	19	42.2
University education	21	46.7
Attendance at sessions		
Session 1	35	77.8
Session 2	33	73.3
Session 3	29	64.4
Session 4	21	46.7
Session 5	31	68.9
Session 6	24	53.3
Completion of questionnaire		
EAT-26	45	100.0
BSQ	45	100.0
NUKYA pre-intervention	44	97.8
NUKYA post-intervention	41	91.1
NUKYA follow-up	35	77.8
Kidmed Index pre-intervention	45	100.0
Kidmed Index follow-up	36	80.0
Completion of diet record		
Pre-intervention	42	93.3
Follow-up	37	82.2
Anthropometric parameters		
Pre-intervention	44	97.8
Follow-up	35	77.8

EAT-26: Eating Attitudes Test, BSQ: Body Shape Questionnaire, NUKYA: Sports Nutrition Knowledge Questionnaire for Young and Adult Athletes.

**Table 4:** Total and sectional scores of sports nutrition knowledge (NUKYA) at pre-intervention, post-intervention, and follow-up.

	Pre Mean (SD)	Post Mean (SD)	Follow-Up Mean (SD)	*p*-Value	Effect Size (Pre/Follow-Up)
	*n* = 44	*n* = 41	*n* = 35		
Total Score	21.1 ^a^ (16.1)	43.1 ^b^ (16.3)	41.4 ^b^ (16.5)	<0.001	1.2
Sections:					
Macronutrients	13.4 ^a^ (10.1)	23.1 ^b^ (10.5)	22.7 ^b^ (9.1)	<0.001	0.9
Micronutrients	7.9 ^a^ (6.2)	10.8 ^ab^ (7.1)	11.6 ^b^ (7.1)	0.005	0.4
Hydration	−0.65 ^a^ (3.8)	6.0 ^b^ (4.2)	4.3 ^b^ (4.1)	<0.001	0.9
Periodization	0.42 ^a^ (2.7)	3.2 ^b^ (2.3)	2.7 ^b^ (3.1)	<0.001	0.7

SD: standard deviation, ^a,b^ In each row, different superscripts indicate statistically significant differences (*p* < 0.05).

**Table 5:** Energy and macronutrient intake at pre-intervention and in the follow-up period (*n* = 36).

	Before		Follow-Up		*p*-Value
	M	SD	M	SD	
Energy (kcal)	2010.4	455.7	1940.2	450.4	0.1620
CHO (g/d)	200.0	44.9	188.0	52.0	0.0802
CHO (% kcal)	40.1	4.8	38.7	5.7	
Fiber (g/d)	17.7	5.4	15.8	6.0	0.0169 *
Protein (g/d)	99.4	27.6	100.0	31.2	0.4449
Protein (% kcal)	19.7	2.7	20.4	3.1	
Lipids (g/d)	90.3	25.3	87.4	20.5	0.2458
Lipids (% kcal)	40.1	4.6	40.9	5.3	
SFA (g/d)	30.3	10.1	30.8	9.9	0.4011
SFA (% kcal)	13.4	3.1	14.2	3.0	
MUFA (g/d)	36.9	12.1	37.1	8.6	0.4632
MUFA (% kcal)	16.2	2.9	17.3	2.3	
PUFA (g/d)	15.5	5.6	12.1	3.2	0.0006 *
PUFA (% kcal)	7.1	2.6	5.8	1.7	
Cholesterol (mg/d)	382.5	161.7	352.2	131.0	0.1235

M: mean; SD: standard deviation; CHO: Carbohydrates; SFA: Saturated fatty acids; MUFA: Monounsaturated fatty acids; PUFA: Polyunsaturated fatty acids. * Significantly different at *p* < 0.05.

**Table 6:** Micronutrient intake pre-intervention and during the follow-up period and percentage of players who do not meet the recommendations (*n* = 36).

	Before		Follow-Up		*p*-Value
	M	SD	M	SD	
Calcium (mg)	856.8	245.0	934.2	375.3	0.0998
Magnesium (mg)	293.8	77.0	288.7	73.3	0.3398
Iron (mg)	13.2	4.5	11.7	3.8	0.0684
Zinc (mg)	9.7	2.5	10.0	3.0	0.2583
Folates (μg)	268.0	109.0	240.5	93.4	0.0713
Vitamin C (mg)	75.8	45.3	83.1	42.1	0.2388
Vitamin D (μg)	3.0	2.5	3.5	2.7	0.5610
Vitamin E (mg **)	10.8	4.3	9.3	2.8	0.0176 *
Vitamin B6 (mg)	2.0	0.6	2.0	0.6	0.3549
Vitamin B12 (μg)	5.7	3.5	5.4	2.5	0.2429

M: mean; SD: standard deviation; * Significantly different at *p* < 0.05; ** Alfatocopherol equivalents.

**Table 7:** Body composition parameters pre-intervention and in the follow-up period (*n* = 35).

	Before		Follow-Up		*p*-Value
	M	SD	M	SD	
Weight (kg)	64.6	9.3	65.4	9.0	0.0175 *
BMI (kg/m^2^)	23.0	2.8	23.3	2.9	0.0161 *
Fat mass (%)	28.4	5.1	29.1	4.7	0.1195
Fat-free mass (%)	71.6	5.1	70.9	4.7	0.1108
Muscle mass (%)	36.2	3.6	35.5	3.4	0.0994

M: mean; SD: standard deviation; BMI: body mass index, * Significantly different at *p* < 0.05.

## Data Availability

The raw data supporting the conclusions of this article will be made available by the authors on request.

## Abbreviations

AGAUR	Agència de Gestió d’Ajuts Universitaris i de Recerca
MD	Mediterranean diet
NUKYA	Sports Nutrition Knowledge Questionnaire for Young and Adult Athletes
EAT-26	Eating Attitudes Test
BSQ	Body Shape Questionnaire
MLM	Mixed linear models
MAR	Missing at random

## References

[1] Janiczak A., Alcock R., Forsyth A., Trakman G.L. (2024). A systematic review of interventions targeting modifiable factors that impact dietary intake in athletes. Br. J. Nutr..

[2] Gastrich M.D., Quick V., Bachmann G., Moriarty A.M. (2020). Nutritional Risks among Female Athletes. J. Women’s Health.

[3] Alcock R., Hislop M., Vidgen H.A., Desbrow B. (2024). Youth and Adolescent Athlete Musculoskeletal Health: Dietary and Nutritional Strategies to Optimise Injury Prevention and Support Recovery. J. Funct. Morphol. Kinesiol..

[4] Birkenhead K.L., Slater G. (2015). A Review of Factors Influencing Athletes’ Food Choices. Sports Med..

[5] Pelly F.E., Thurecht R.L., Slater G. (2022). Determinants of Food Choice in Athletes: A Systematic Scoping Review. Sports Med.-Open.

[6] Spronk I., Kullen C., Burdon C., O’Connor H. (2014). Relationship between nutrition knowledge and dietary intake. Br. J. Nutr..

[7] Janiczak A., Devlin B.L., Forsyth A., Trakman G.L. (2022). A systematic review update of athletes’ nutrition knowledge and association with dietary intake. Br. J. Nutr..

[8] Mcmanus A.M., Armstrong N., Armstrong N., Mcmanus A.M. (2011). Physiology of Elite Young Female Athletes. The Elite Young Athlete; Medicine and Sport Science.

[9] Sims S.T., Kerksick C.M., Smith-Ryan A.E., Janse de Jonge X.A.K., Hirsch K.R., Arent S.M., Hewlings S.J., Kleiner S.M., Bustillo E., Tartar J. (2023). International society of sports nutrition position stand: Nutritional concerns of the female athlete. J. Int. Soc. Sports Nutr..

[10] Veloso-Pulgar M., Farran-Codina A., Fernández de Arriba R. (2025). Effects of nutrition education programs designed to improve dietary intake and nutritional knowledg. in female athletes: A systematic review. Nutr. Res. Rev..

[11] Aguilo A., Lozano L., Tauler P., Nafría M., Colom M., Martínez S. (2021). Nutritional status and implementation of a nutritional education program in young female artistic gymnasts. Nutrients.

[12] Tektunalı Akman C., Gönen Aydın C., Ersoy G. (2024). The effect of nutrition education sessions on energy availability, body composition, eating attitude and sports nutrition knowledge in young female endurance athletes. Front. Public Health.

[13] Condo D., Lohman R., Kelly M., Carr A. (2019). Nutritional Intake, Sports Nutrition Knowledge and Energy Availability in Female Australian Rules Football Players. Nutrients.

[14] Aparicio-Martinez P., Perea-Moreno A.-J., Martinez-Jimenez M.P., Redel-Macías M.D., Pagliari C., Vaquero-Abellan M. (2019). Social Media, Thin-Ideal, Body Dissatisfaction and Disordered Eating Attitudes: An Exploratory Analysis. Int. J. Environ. Res. Public Health.

[15] Black K.E., Baker D.F., Sims S.T. (2020). Nutritional Needs of the Female Athlete: Risk and Prevention of Low Energy Availability. Strength Cond. J..

[16] Hopper C., Mooney E., Mc Cloat A. (2025). Nutritional Intake and Dietary Knowledge of Athletes: A Scoping Review. Nutrients.

[17] Vázquez-Espino K., Rodas-Font G., Farran-Codina A. (2022). Sport Nutrition Knowledge, Attitudes, Sources of Information, and Dietary Habits of Sport-Team Athletes. Nutrients.

[18] Heikkilä M., Lehtovirta M., Autio O., Fogelholm M., Valve R. (2019). The impact of nutrition education intervention with and without a mobile phone application on nutrition knowledge among young endurance athletes. Nutrients.

[19] Manore M.M., Patton-Lopez M.M., Meng Y., Wong S.S. (2017). Sport nutrition knowledge, behaviors and beliefs of high school soccer players. Nutrients.

[20] Martinez-Lacoba R., Pardo-Garcia I., Amo-Saus E., Escribano-Sotos F. (2018). Mediterranean diet and health outcomes: A systematic meta-review. Eur. J. Public Health.

[21] Griffiths A., Matu J., Whyte E., Akin-Nibosun P., Clifford T., Stevenson E., Shannon O.M. (2022). The Mediterranean dietary pattern for optimising health and performance in competitive athletes: A narrative review. Br. J. Nutr..

[22] Bianchi E., Erbasan H., Riso P., Perna S. (2024). Impact of the Mediterranean Diet on Athletic Performance, Muscle Strength, Body Composition, and Antioxidant Markers in Both Athletes and Non-Professional Athletes: A Systematic Review of Intervention Trials. Nutrients.

[23] Murimi M.W., Kanyi M., Mupfudze T., Amin M.R., Mbogori T., Aldubayan K. (2017). Factors Influencing Efficacy of Nutrition Education Interventions: A Systematic Review. J. Nutr. Educ. Behav..

[24] Vázquez-Espino K., Fernández-Tena C., Lizarraga-Dallo M.A., Farran-Codina A. (2020). Development and validation of a short sport nutrition knowledge questionnaire for athletes. Nutrients.

[25] Rivas T., Bersabé R., Jiménez M., Berrocal C. (2010). The Eating Attitudes Test (EAT-26): Reliability and validity in Spanish female samples. Span. J. Psychol..

[26] Dowson J., Henderson L. (2001). The validity of a short version of the Body Shape Questionnaire. Psychiatry Res..

[27] Serra-Majem L., Ribas L., Ngo J., Ortega R.M., García A., Pérez-Rodrigo C., Aranceta J. (2004). Food, youth and the Mediterranean diet in Spain. Development of KIDMED, Mediterranean Diet Quality Index in children and adolescents. Public Health Nutr..

[28] Ramírez-Contreras C., Farran-Codina A., Zerón-Rugerio M.F., Izquierdo-Pulido M. (2023). Relative Validity and Reliability of the Remind App as an Image-Based Method to Assess Dietary Intake and Meal Timing in Young Adults. Nutrients.

[29] Casperson S.L., Sieling J., Moon J., Johnson L., Roemmich J.N., Whigham L. (2015). A mobile phone food record app to digitally capture dietary intake for adolescents in a free-living environment: Usability study. JMIR Mhealth Uhealth.

[30] Cantos D., Farran-Codina A., Palma-Linares I. (2013). Programa de Càlcul Nutricional PCN Pro Versió 1. https://diposit.ub.edu/dspace/handle/2445/44329?locale=es.

[31] Codina A.F., de Arriba R.F., González A.G., Almiñana S.J. (2022). Tablas Estándar de Composición de Los Alimentos–TECA: Taules Estàndard de Composició Dels Aliments-TECA.

[32] Ruiz-López M.D., Reyes Artacho M.L. (2011). Guía para Estudios Dietéticos. Álbum Fotográfico de Alimentos.

[33] Ruiz-López M.D., Ruiz-López M.D., Martínez de Victoria Muñoz E., Gil Hernández A. (2019). Martínez de Victoria Muñoz, E; Gil Hernández, A. Guía Fotográfica de Porciones de Alimentos Consumidos en España.

[34] Wiita B.G., Stombaugh I.A. (1996). Nutrition Knowledge, Eating Practices, and Health of Adolescent Female Runners: A 3-Year Longitudinal Study. Int. J. Sport Nutr..

[35] Davar V. (2012). Nutritional Knowledge and Attitudes Towards Healthy Eating of College-going Women Hockey Players. J. Hum. Ecol..

[36] Abood D.A., Black D.R., Birnbaum R.D. (2004). Nutrition education intervention for college female athletes. J. Nutr. Educ. Behav..

[37] Chapman P., Toma R.B., Tuveson R.V., Jacob M. (1997). Nutrition Knowledge among Adolescent High School Female Athletes. Adolescence.

[38] Gonçalves C.B., Nogueira J.A.D., da Costa T.H.M. (2014). The Food Pyramid Adapted to Physically Active Adolescents as a nutrition education tool. Rev. Bras. Ciências Esporte.

[39] Patton-Lopez M.M., Manore M.M., Branscum A., Meng Y., Wong S.S. (2018). Changes in sport nutrition knowledge, attitudes/beliefs and behaviors following a two-year sport nutrition education and life-skills intervention among high school soccer players. Nutrients.

[40] Torres-McGehee T.M., Green J.M., Leaver-Dunn D., Leeper J.D., Bishop P.A., Richardson M.T. (2011). Attitude and Knowledge Changes in Collegiate Dancers following a Short-Term, Team-Centered Prevention Program on Eating Disorders. Percept. Mot. Ski..

[41] Collison S.B. (1996). Impact of Nutrition Education on Female Athletes. Am. J. Health Behav..

[42] Daniel N.V.S., Jürgensen L.P., Padovani R.D.C., Juzwiak C.R. (2016). Impact of an interdisciplinary food, nutrition and health education program for adolescent Brazilian volleyball players. Rev. Nutr..

[43] Kunkel M.E., Bell L.B., Luccia B.H.D. (2001). Peer Nutrition Education Program to Improve Nutrition Knowledge of Female Collegiate Athletes. J. Nutr. Educ..

[44] Laramée C., Drapeau V., Valois P., Goulet C., Jacob R., Provencher V., Lamarche B. (2017). Evaluation of a Theory-Based Intervention Aimed at Reducing Intention to Use Restrictive Dietary Behaviors Among Adolescent Female Athletes. J. Nutr. Educ. Behav..

[45] Lydon R., McCloat A., Mooney E., Kelly-Blakeney E. (2023). Recipes for Success: Lessons learned from the implementation of a food skills and nutrition education workshop with Gaelic athletic players on the Island of Ireland (IOI). Health Educ. J..

[46] Martinelli L. (2013). The implementation and evaluation of a nutrition education programme for university elite athletes. Prog. Nutr..

[47] Valliant M.W., Emplaincourt H.P., Wenzel R.K., Garner B.H. (2012). Nutrition education by a registered dietitian improves dietary intake and nutrition knowledge of a NCAA female volleyball team. Nutrients.

[48] Zaman N.N.Z.K., Muhamad A.S., Jusoh M.R.C. (2021). Knowledge, attitude, practice (KAP) and dietary intake of young university athletes following sports nutrition education. Malays. J. Nutr..

[49] Yannakoulia M., Sitara M., Matalas A.L. (2002). Reported eating behavior and attitudes improvement after a nutrition intervention program in a group of young female dancers. Int. J. Sport Nutr. Exerc. Metab..

[50] Trakman G.L., Forsyth A., Hoye R., Belski R. (2019). Australian team sports athletes prefer dietitians, the internet and nutritionists for sports nutrition information. Nutr. Diet..

[51] Wardle J., Parmenter K., Waller J. (2000). Nutrition knowledge and food intake. Appetite.

[52] Hamilton Greg J., Thomson Christine D., Hopkins William G. (1994). Nutrition knowledge of elite distance runners. N. Z. J. Sports Med..

[53] Sofi F., Macchi C., Abbate R., Gensini G.F., Casini A. (2014). Mediterranean diet and health status: An updated meta-analysis and a proposal for a literature-based adherence score. Public Health Nutr..

[54] Kaufman M., Nguyen C., Shetty M., Oppezzo M., Barrack M., Fredericson M. (2023). Popular Dietary Trends’ Impact on Athletic Performance: A Critical Analysis Review. Nutrients.

[55] D’Angelo S. (2020). Polyphenols: Potential beneficial effects of these phytochemicals in athletes. Curr. Sports Med. Rep..

[56] Lewis N.A., Daniels D., Calder P.C., Castell L.M., Pedlar C.R. (2020). Are There Benefits from the Use of Fish Oil Supplements in Athletes? A Systematic Review. Adv. Nutr..

[57] Bifulco M., Cerullo G., Abate M. (2019). Is the Mediterranean Diet Pattern a Good Choice for Athletes?. Nutr. Today.

[58] Philippou E., Middleton N., Pistos C., Andreou E., Petrou M. (2017). The impact of nutrition education on nutrition knowledge and adherence to the Mediterranean Diet in adolescent competitive swimmers. J. Sci. Med. Sport.

[59] Sahnoune R., Bouchenak M. (2020). Nutritional intervention promoting Mediterranean diet improves dietary intake and enhances score adherence in adolescent athletes. Mediterr. J. Nutr. Metab..

[60] Veloso-Pulgar M., Arcila-Agudelo A.M., Ferrer-Svoboda C., Torres-Fernández T., Farran-Codina A. (2024). Estado nutricional y adherencia a la dieta mediterránea en la población escolar de la ciudad de Mataró (Cataluña, España). Nutr. Hosp..

[61] Nowacka E., Leszczyńska T., Kopeć A., Hojka D. (2016). Nutritional behavior of Polish canoeist’s athletes: The interest of nutritional education. Sci. Sports.

[62] Saenz C., Sanders D.J., Brooks S.J., Bracken L., Jordan A., Stoner J., Vatne E., Wahler M., Brown A.F. (2024). TRelationship Between Dance Training Volume, Body Composition, and Habitual Diet in Female Collegiate Dancers: The Intercollegiate Artistic Athlete Research Assessment (TIAARA) Study. Nutrients.

[63] Manore M.M. (1996). Chronic dieting in active women: What are the health consequences?. Women’s Health Issues.

[64] Thomas D.T., Erdman K.A., Burke L.M. (2016). American College of Sports Medicine Joint Position Statement. Nutrition and Athletic Performance. Med. Sci. Sports Exerc..

[65] Holtzman B., Ackerman K.E. (2021). Recommendations and Nutritional Considerations for Female Athletes: Health and Performance. Sports Med..

[66] Larrosa M., Gil-Izquierdo A., González-Rodríguez L.G., Alférez M.J.M., San Juan A.F., Sánchez-Gómez Á., Calvo-Ayuso N., Ramos-Álvarez J.J., Fernández-Lázaro D., Lopez-Grueso R. (2025). Nutritional Strategies for Optimizing Health, Sports Performance, and Recovery for Female Athletes and Other Physically Active Women: A Systematic Review. Nutr. Rev..

[67] de la Sociedad Española G.C., de Nutrición Comunitaria S.E.N.C. (2016). Guías alimentarias para la población española (SENC, diciembre 2016): La nueva pirámide de la alimentación saludable. Nutr. Hosp..

[68] Barney D.E., Cheung S.N., Harris A.R., Berryman C.E., Hennigar S.R. (2024). Dietary Intake and Diet Quality of Female and Male NCAA Division I Cross Country Runners from a Single University. Curr. Dev. Nutr..

[69] Lagowska K., Kapczuk K., Jeszka J. (2014). Nine-month nutritional intervention improves restoration of menses in young female athletes and ballet dancers. J. Int. Soc. Sports Nutr..

[70] Lagowska K., Kapczuk K., Friebe Z., Bajerska J. (2014). Effects of dietary intervention in young female athletes with menstrual disorders. J. Int. Soc. Sports Nutr..

[71] Castillo M., Lozano-Casanova M., Sospedra I., Norte A., Gutiérrez-Hervás A., Martínez-Sanz J.M. (2022). Energy and Macronutrients Intake in Indoor Sport Team Athletes: Systematic Review. Nutrients.

[72] Jeukendrup A.E. (2014). High-carbohydrate versus high-fat diets in endurance sports. Schweiz. Z. Sportmed. Sport..

[73] Serra M.C., Beavers K.M., Ziegenfuss T. (2015). Essential and Nonessential Micronutrients and Sport. Nutritional Supplements in Sports and Exercise.

[74] Potgieter S. (2013). Sport nutrition: A review of the latest guidelines for exercise and sport nutrition from the American College of Sport Nutrition, the International Olympic Committee and the International Society for Sports Nutrition: Review article. S. Afr. J. Clin. Nutr..

[75] Pingitore A., Pace G., Lima P., Mastorci F., Quinones A., Iervasi G., Vassalle C. (2015). Exercise and oxidative stress: Potential effects of antioxidant dietary strategies in sports. Nutrition.

[76] Estudio ENALIA 2012–2014 (2017). Encuesta Nacional de consumo de Alimentos en población Infantil y Adolescente.

[77] World Health Organization (2003). Diet, Nutrition and the Prevention of Chronic Diseases: Report of a Joint WHO/FAO Expert Consultation.

[78] Campa F., Toselli S., Mazzilli M., Gobbo L.A., Coratella G. (2021). Assessment of Body Composition in Athletes: A Narrative Review of Available Methods with Special Reference to Quantitative and Qualitative Bioimpedance Analysis. Nutrients.

[79] Anderson D.E. (2010). The Impact of Feedback on Dietary Intake and Body Composition of College Women Volleyball Players Over a Competitive Season. J. Strength Cond. Res..

[80] Terenzio A., Cassera A., Gervasoni A., Pozzi A., Orlando A., Greco A., Palestini P., Cazzaniga E. (2021). The impact of a nutritional intervention program on eating behaviors in Italian athletes. Int. J. Environ. Res. Public Health.

[81] Wenzel R.K., Valliant M.W., Chang Y., Bomba A.K., Lambert L.G. (2012). Dietary Assessment and Education Improves Body Composition and Diet in NCAA Female Volleyball Players. Top. Clin. Nutr..

[82] Torres-McGehee T.M., Pritchett K.L., Zippel D., Minton D.M., Cellamare A., Sibilia M. (2012). Sports nutrition knowledge among collegiate athletes, coaches, athletic trainers, and strength and conditioning specialists. J. Athl. Train..

[83] Schoeller D.A., Bandini L.G., Dietz W.H. (1990). Inaccuracies in self-reported intake identified by comparison with the doubly labelled water method. Can. J. Physiol. Pharmacol..

[84] Hand R.K., Abram J.K., Brown K., Ziegler P.J., Parrott J.S., Steiber A.L. (2015). Development and Validation of the Guide for Effective Nutrition Interventions and Education (GENIE): A Tool for Assessing the Quality of Proposed Nutrition Education Programs. J. Nutr. Educ. Behav..

